# Non-FFP-Based Magnetic Particle Imaging (NFMPI) with an Open-Type RF Coil System: A Feasibility Study

**DOI:** 10.3390/s25030665

**Published:** 2025-01-23

**Authors:** Chan Kim, Jiyun Nan, Kim Tien Nguyen, Jong-Oh Park, Eunpyo Choi, Jayoung Kim

**Affiliations:** 1 Korea Institute of Medical Microrobotics, Gwangju 61186, Republic of Korea; kc9134@kimiro.re.kr (C.K.); nguyenkimtien90@gmail.com (K.T.N.); jop@kimiro.re.kr (J.-O.P.); 2 School of Mechanical Engineering, Chonnam National University, Gwangju 61186, Republic of Korea; njy_kiwoon@kimiro.re.kr; 3 Department of Biosystems Engineering, Chungbuk National University, Cheongju 28644, Republic of Korea

**Keywords:** targeted drug delivery, magnetic particle imaging, non-FFP-based method, open-type MPI scanner

## Abstract

Active drug delivery systems for cancer therapy are gaining attention for their biocompatibility and enhanced efficacy compared to conventional chemotherapy and surgery. To improve precision in targeted drug delivery (TDD), actuating devices using external magnetic fields are employed. However, a key challenge is the inability to visually track magnetic drug carriers in blood vessels, complicating navigation to the target. Magnetic particle imaging (MPI) systems can localize magnetic carriers (MCs) but rely on bulky electromagnetic coils to generate a static magnetic field gradient, creating a field-free point (FFP) within the field of view (FOV). Also, additional coils are required to move the FFP across the FOV, limiting flexibility and increasing the system size. To address these issues, we propose a non-FFP-based, open-type RF coil system with a simplified structure composed of a Tx/Rx coil and a permanent magnet at the coil center, eliminating the need for an FFP. Furthermore, integrating a robotic arm for coil assembly enables easy adjustment of the FOV size and location. Finally, imaging tests with magnetic nanoparticles (MNPs) confirmed the system’s ability to detect and localize a minimum mass of 0.3 mg (Fe) in 80 × 80 mm^2^.

## 1. Introduction

Targeted drug delivery (TDD) has been considered an effective therapeutic solution to achieve better outcomes in cancer treatment. Conventional cancer treatment methods, such as chemotherapy or traditional incision surgery to remove tumors, have side effects such as overdoses of toxic drugs, infection, and tissue damage [1,2,3,4]. On the other hand, TDD is a non-invasive treatment method that injects small amounts of drugs into blood vessels and actively directs them to a targeted lesion [4,5]. Due to its promising features, advanced studies have recently been conducted to apply micrometer-sized robots as drug-loaded carriers to increase therapeutic performances through the TDD systems [6,7]. The microrobots can carry therapeutic agents faster by external sources of the magnetic field, ultrasound and light, which can be supportive components in the drug delivery process [8,9,10]. In particular, microrobots based on magnetic nanoparticles (MNPs) can be guided with a superior access speed and release drugs into a targeted tumor site with high efficiency [11]. Electromagnetic systems generating well-designed magnetic fields in a region of interest (ROI) can be utilized for a precise magnetic actuation [12,13,14]. Magnetic-based localization systems can enhance TDD performance by providing position information on the microrobots in the body for accurate guidance to a target lesion. Despite the precise manipulation of magnetic nanoparticles (MNPs) to a targeted position, the percentage of drug carriers reaching the intended site varies due to factors such as particle size, vessel dimensions, and the depth between the boundary of the generated magnetic field and the targeted area. Therefore, the localization system must account for these medical and physiological conditions to effectively detect smaller volumes of MNPs.

Magnetic particle imaging (MPI) is a promising technique for visualizing the distribution of magnetic particles and has been newly devised since it was first proposed in 2005 [15]. MPI can achieve imaging performance with high temporal and spatial resolutions based on the response of MNPs induced by static and alternating magnetic fields [16]. In an MPI system, MNPs are excited within a range of inherent saturation point owing to their magnetization characteristics. The excited level of MNPs has an impact on changing the response signal of MNPs that is described as imaging pixels [17]. In general, conventional MPI techniques employ specific magnetic field forms, called a field-free point (FFP) or a field-free line (FFL), to adjust the magnetization level of MNPs [18,19,20,21]. FFP and FFL mean a point and a line where the magnetic field is zero, respectively, and the MPI systems were designed to create them in symmetrically arranged structures of electromagnets and permanent magnets. Furthermore, bore-type radio frequency (RF) systems consisting of a transmitting coil (Tx coil) and a receiving coil (Rx coil) were embedded in the MPI systems to stably acquire the response signals of MNPs. In recent advancements in MPI systems, P. Vogel et al. introduced a novel portable interventional MPI (iMPI) system, presenting a promising approach for applying MPI technology in clinical settings. The iMPI system employs a bore-type design and is specifically tailored for imaging human limbs, with a primary emphasis on creating a clinically viable MPI system for leg imaging. In their study, the iMPI system successfully visualized vascular pathologies such as aneurysms and stenosis, enabling real-time guidance for percutaneous transluminal angioplasties [22].

Another significant development is reported by Florian Thieben et al., who designed an MPI system specifically for brain imaging. This system shows considerable potential for diagnosing and monitoring neurovascular conditions, including ischemic stroke, intracranial hemorrhage, and traumatic brain injuries. The researchers validated the system’s dynamic imaging accuracy and resolution by simulating cerebral perfusion and utilizing models with varying degrees of stenosis [23]. Collectively, these studies suggest that MPI technology is gradually advancing toward clinical applications in humans in various forms, demonstrating great potential for future development. However, these enclosed geometries of the MPI systems make it difficult to expand the workspace into human-scale medical coverage, hence they have been limited to animal applications exclusively. Therefore, structural improvements are required to reach the ultimate purpose of MPI for human applications. 

This study, to handle the issues, focuses on developing an open-structured RF system capable of imaging MNPs. In the literature, there have been attempts to build an open-type RF system using a Tx-Rx combined coil of a static circular structure [24] and a hand-held cylindrical structure [25]. However, the main function of the RF systems is only to detect MNPs, and they unable to image and localize MNPs inside the human body, since FFPs and FFLs cannot be produced without additional magnetic sources in the RF systems. Therefore, we propose a novel method for MPI without FFPs and FFLs using an open-type RF system devised in this study. The proposed method focuses on image visualization using a negative signal of MNPs responded to a static magnetic field, unlike using a positive signal in the conventional RF systems. To accomplish it, an open-type RF system is developed by containing a cylindrical permanent magnet inside RF coils that generates the static magnetic field for magnetic saturation of MNPs instead of creating FFPs and FFLs. In addition, the devised RF system is manipulated by an articulated robot-arm to collect the signal of MNPs in a workspace. Finally, MPI performance is validated via MNP imaging tests in 2D and 3D phantom environments.

## 2. Open-Type RF System for Non-FFP-Based Magnetic Particle Imaging (NFMPI)

### 2.1. Open-Type RF System Configuration

**Figure 1:**
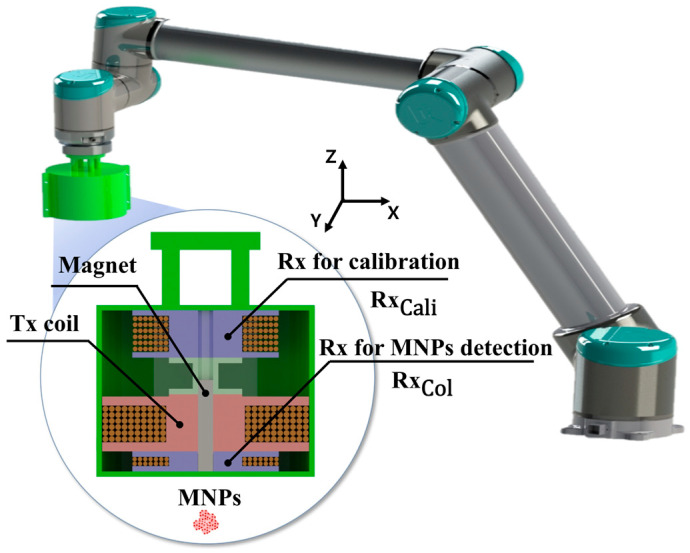
Configuration diagram of a non-FFP-based MPI system (NFMPI). NFMPI consists of three coils (one Tx coil and two Rx coils) and a cylindrical permanent magnet. Based on the center magnet, the Tx coil is placed between the Rx coils to create an alternating magnetic field (AMF), while the Rx coils are placed at the top and bottom of the Tx coil to detect the MNP signal (Rx_col_) and to reduce the residual signal of the RF system (Rx_cali_), respectively.

The conceptual prototype of the localization coil assembly shown in Figure 1 consists of a Tx coil, a Rx coil divided into a coil for calibration in Rx (${\mathrm{Rx}}_{\mathrm{Cali}}$) and for collecting signals of MNPs (${\mathrm{Rx}}_{\mathrm{Col}}$), and a permanent magnet positioned in a center of Tx/Rx coils. The ${\mathrm{Rx}}_{\mathrm{Cali}}$ and ${\mathrm{Rx}}_{\mathrm{Col}}$ are connected as one receiving coil chain. The assembly of coils is fixed to the edge of a robot arm to be controlled along the designated trajectory in an ROI to scan MNPs. 

The contents of specifications of coils are summarized in Table 1. A magnet at the center of the coil assembly is a cylinder type with a diameter of 8 mm and a height of 50 mm. In case of an imperfect experimental setup, such as an unstable alternating magnetic field (AMF) of Tx coil, induced voltage signals recorded in the Rx coil include a noise. To minimize the amplitude level of voltage signals in a non-MNPs case, ${\mathrm{Rx}}_{\mathrm{Cali}}$ wound reversely to ${\mathrm{Rx}}_{\mathrm{Col}}$ was employed. Assuming the ${\mathrm{Rx}}_{\mathrm{Cali}}$, ${\mathrm{Rx}}_{\mathrm{Col}}$ and Tx coil are aligned in the *z*-axis shown in Figure 1, the center position of ${\mathrm{Rx}}_{\mathrm{Cali}}$ and ${\mathrm{Rx}}_{\mathrm{Col}}$ based on the center of the Tx coil can be defined as $P_{R_{\mathrm{Cali}}}$ and $P_{R_{\mathrm{Col}}}$. When the Tx coil generates AMF, ${\mathrm{Rx}}_{\mathrm{Cali}}$ and ${\mathrm{Rx}}_{\mathrm{Col}}$ are inducted magnetically. Then, the magnetic flux passing on the inner area of ${\mathrm{Rx}}_{\mathrm{Cali}}$ and ${\mathrm{Rx}}_{\mathrm{Col}}$ can be defined as follows:

(1) $$\Phi_{R_{\mathrm{Cali}}}=\iint_{A}-B_{Tx}\left(P_{R_{\mathrm{Cali}}}\right)\cdot dA_{R_{\mathrm{Cali}}}$$

(2) $$\Phi_{R_{\mathrm{Col}}}=\iint_{A}B_{Tx}\left(P_{R_{\mathrm{Col}}}\right)\cdot dA_{R_{\mathrm{Col}}}$$

where $B_{Tx}(P_{R_{\mathrm{Cali}}}$) and $B_{Tx}(P_{R_{\mathrm{Col}}}$) denotes the magnetic flux density on the *z*-axis by the Tx coil at the positions of $P_{R_{\mathrm{Cali}}}$ and $P_{R_{\mathrm{Col}}}$. $A_{R_{\mathrm{Cali}}}$ and $A_{R_{\mathrm{Col}}}$ are the inner area of each Rx coil. The induced voltage by magnetic fluxes on ${\mathrm{Rx}}_{\mathrm{Cali}}$ and ${\mathrm{Rx}}_{\mathrm{Col}}$ can be expressed as

(3) $$V_{R_{\mathrm{Cali}}}=-N_{R_{\mathrm{Cali}}}\frac{d\Phi_{R_{\mathrm{Cali}}}}{dt}$$

(4) $$V_{R_{\mathrm{Col}}}=-N_{R_{\mathrm{Col}}}\frac{d\Phi_{R_{\mathrm{Col}}}}{dt}$$

where $N_{R_{\mathrm{Cali}}}$ and $N_{R_{\mathrm{Col}}}$ means the number of turns of ${\mathrm{Rx}}_{\mathrm{Cali}}$ and ${\mathrm{Rx}}_{\mathrm{Col}}$. Finally, the inductive voltage in Rx coils can be attained by the combination of $V_{R_{\mathrm{Cali}}}$ and $V_{R_{\mathrm{Col}}}$ as

(5) $$V_{res}=V_{R_{\mathrm{Cali}}}+V_{R_{\mathrm{Col}}}$$

where $V_{res}$ presents the residual voltage in Rx coils. Moreover, to attenuate $V_{res}$ to be minimized, the vertical distance of each Rx coil from the center of the Tx coil can be manipulated. In this system, we set up a ${\mathrm{Rx}}_{\mathrm{Col}}$ connected on the bottom of the Tx coil, as shown in Figure 1, to move ${\mathrm{Rx}}_{\mathrm{Cali}}$ along the *z*-axis to measure $V_{res}$. Also, we increased the turns of ${\mathrm{Rx}}_{\mathrm{Cali}}$ three times more than ${\mathrm{Rx}}_{\mathrm{Col}}$ to drop $V_{res}$ sharply, depending on the distance from the Tx coil. For the measurement tests, one ampere (1A) was transmitted to the Tx coil. In the tests, the receiving circuit showed the minimum $V_{res}$ (0.12 V) in the distance of 58 mm from the center of the Tx coil. To maintain the optimized distance, a frame structure was designed and printed to connect ${\mathrm{Rx}}_{\mathrm{Cali}}$ with the Tx coil.

### 2.2. Non-FFP-Based MPI Method

**Figure 2:**
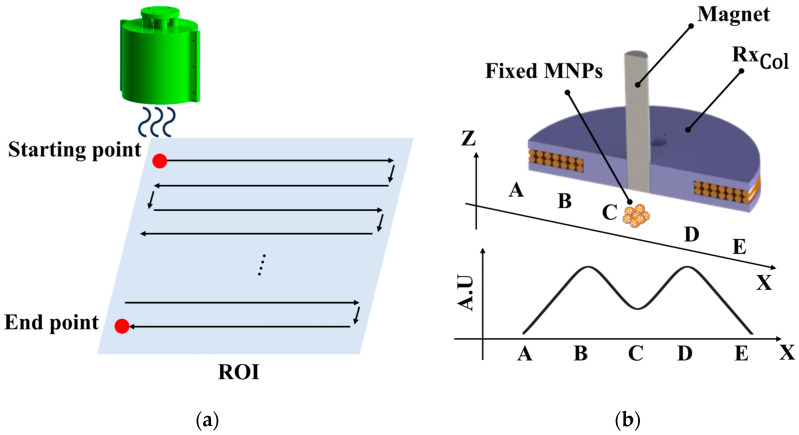
Conceptual diagram of the non-FFP-based magnetic particle imaging. (**a**) Scanning trajectory on the ROI plane. (**b**) Continuous magnetic particle signal induced by the receiving coil from A to E when the MNP group is at C.

The trajectory of a localization system can vary by changing the path generation coding. Figure 2 illustrates a method of scanning MNPs where a coil system moves along a defined path and measures signals during a procedure of scanning. In this paper, as depicted in Figure 2a, a zigzag path was employed to scan MNPs. Assuming MNPs are fixed at the point C (the center of ${\mathrm{Rx}}_{\mathrm{Col}}$) in Figure 2b, the coil system passes over it from A to E along the *x*-axis. When ${\mathrm{Rx}}_{\mathrm{Col}}$ moves from A to B, the AMF of the Tx coil induces a random rotation of MNPs, which makes a ${\mathrm{Rx}}_{\mathrm{Col}}$-detecting signal for MNPs. In the movement of the coil system from B to C, the magnetic field by the magnet affects the MNPs, making them saturated so that the signal of the MNPs is reduced. The movements from C to D and D to E are identically applied to those from A to B and B to C. Finally, a signal graph of MNPs includes two positive peaks and one negative peak. In this paper, we identify the negative peak as a valley point, indicating the predicted position of MNPs.

**Figure 3:**
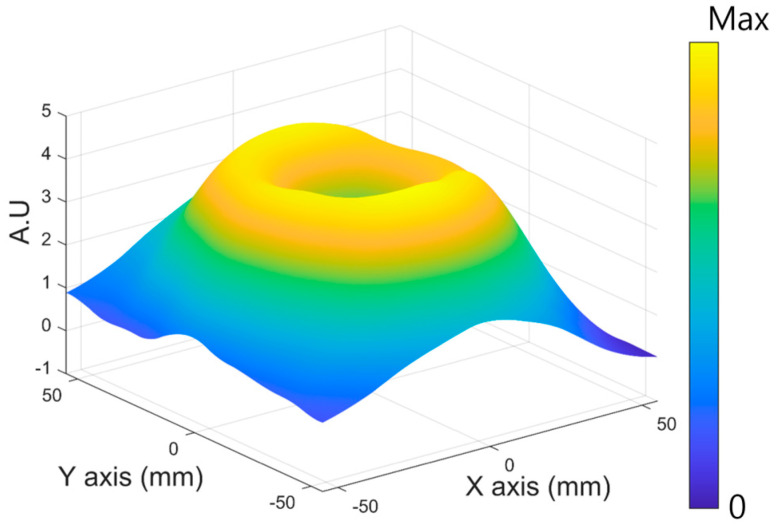
A three-dimensional visualization of the received magnetic particle signal on the XY plane. The received magnetic particle signal is distributed in the form of a mountain basin according to the change in particle magnetization caused by the static magnetic field of the permanent magnet.

**Figure 4:**
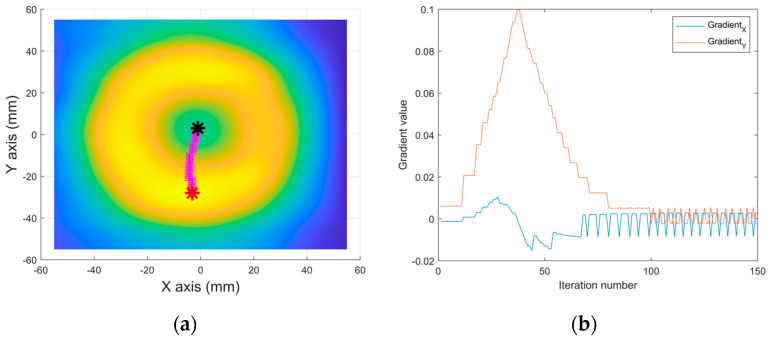

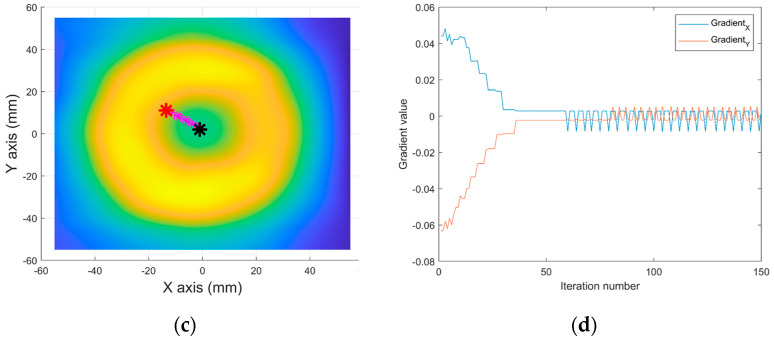
A gradient descent method to find a valley point on the measured data image. (**a**) A 2D image on the XY plane, including a red star displaying a starting point as a peak point of image, a black star displaying a final valley point, and pink stars displaying calculated locations by the gradient descent method between a starting point and a valley point. (**b**) a Gradient value graph during the iteration process of (**a**). (**c**) Finding a valley point from a starting point manually picked by an operator. (**d**) A gradient value graph during the iteration process of (**c**).

Assuming that the concentration of MNPs is located at the center of the ROI, the MPI system conducts scanning along the trajectory. Figure 3 shows a sample of a resultant graph in the case of scanning MNPs at (0,0) on the XY plane. As expected, a valley point is located adjacent to the central area. To specify the location of the valley point, we employed the conventional gradient descent method (GDM) [26]. The GDM has been utilized in deep neural networks to find a global minimum point of acquired data. The valley point in the measured image of MNPs exhibits a similar property to a global minimum point. When we set the starting point on the XY plane as P($x_{0}$, $y_{0}$), the location of the point can be defined as

(6) $${P(x}_{i},y_{i})={P(x}_{i-1},y_{i-1})-\alpha *\nabla P(x_{i-1},y_{i-1})$$

where α is the learning rate and i is the iteration number. If i is large enough to arrive at the valley point, the gradient value of that point is approximately close to zero $\nabla P\left(x_{i-1},y_{i-1}\right)\to 0$. In Figure 4, the learning rate and the iteration number were chosen as 10 and 150. Both methods used in Figure 4a,c calculated the same identical location for the valley point as (−1, 3). For the actual application of the GDM into the MPI system, the starting point is automatically and randomly selected as one of the positive peaks in order to go in search of the valley point.

## 3. Experimental Results

### 3.1. Experimental Setup

**Figure 5:**
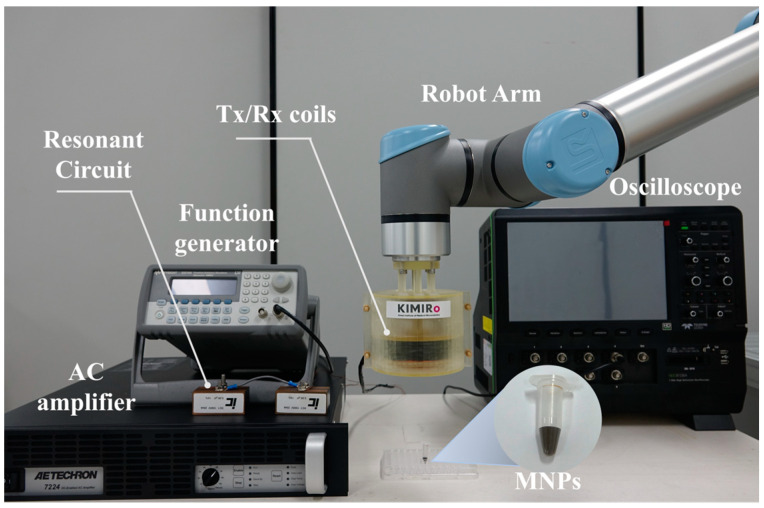
Experimental setup of the NFMPI system.

**Figure 6:**
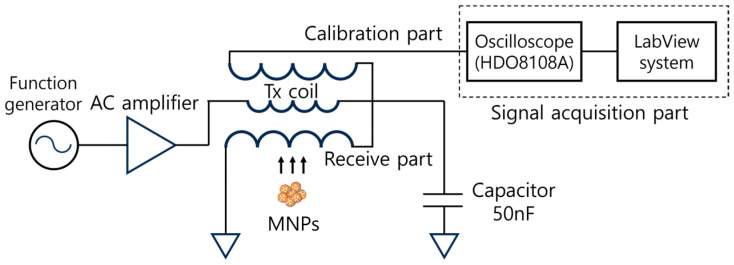
Circuit diagram of the NFMPI system.

For validating the localization performance, the proposed system was set up as shown in Figure 5 and Figure 6. A function generator (KEYSIGHT 33210A, Keysight Technologies, Santa Rosa, CA, USA) transfers alternating signals to an AC amplifier (AE Techron 7224, AE Techron, Elkhart, IN, USA). Amplified signals transmit to the resonant circuit of the Tx coils. The targeted resonance frequency is 5000 Hz. The measured capacitor volume by the LCR meter (HIOKI 3522–50 LCR HiTESTER, MCi Globaltronics Pte Ltd., Singapore) that was set to match the frequency was 50 nF. Measured signals in the Rx chain are transferred to oscilloscope (Teledyne Lecroy HDO8108A, Avalon Test Equipment, Carlsbad, CA, USA). Including a band pass filter (4500 Hz to 5500 Hz), LabView (National Instrument, Austin, TX, USA) program records the final signal in real time. Synomag (Gentaur Genprice 104–00–701, Micromod GmbH, Rostock, Germany) is utilized to source MNPs for the detection and localization experiments. The average iron content of Synomag is 10 mg/mL. The robotic arm (UR10e, Universal Robot, Odense, Denmark) with six degrees of freedom was used to make a scanning trajectory along the designated path on the three-dimensional space.

### 3.2. One-Dimensional Detection Performance

**Figure 7:**
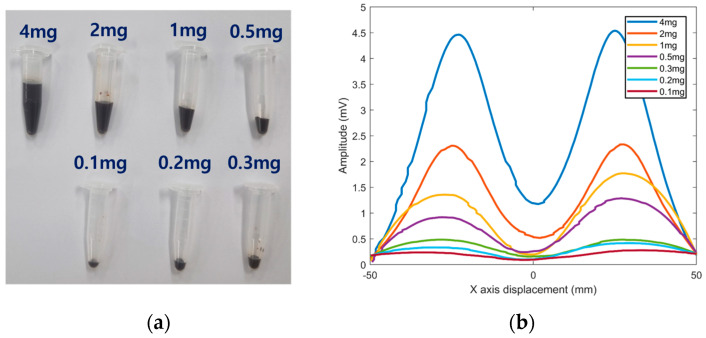
1D analysis of received signals depending on various amounts of MNP.

Experiments for one-dimensional detection along the *x*-axis were conducted using the proposed system. The iron contents of MNPs were ranged from 4 mg to 0.1 mg, as shown in Figure 7a. The position of the MNP container was fixed on the center of the *x*-axis. The distance between the coils and MNPs on the *z*-axis was set to 25 mm to achieve a sufficient magnetic saturation of MNPs up to 11 mT. The sampling time for the acquisition of MNP response signals was 50 ms. The robotic arm was controlled to move coils continuously with a velocity of 6 mm/s and the input current to Tx coil was 5 A. The measured results for each case are illustrated in Figure 7b. To obtain a more accurate valley point, a data smoothing algorithm was employed to remove the noise of the raw signal data. The depths between the averages of the two positive peak points and the predicted valley points across the seven cases from the mass of 4 mg to 0.1 mg were measured as 3.3 mV, 1.8 mV, 1.3 mV, 0.8 mV, 0.3 mV, 0.2 mV, and 0.1 mV, respectively. In correlation with reducing the mass of the iron content, the depth of the valley and the amplitude of the positive peaks were decreased linearly. As a result, the MNP signals were evaluated, indicating that the measurable minimum MNP mass to form the valley was 0.3 mg; moreover, the feasibility of the proposed system to detect the location of MNPs by up to 0.3 mg was demonstrated.

### 3.3. Two-Dimensional Localization Performance

**Figure 8:**
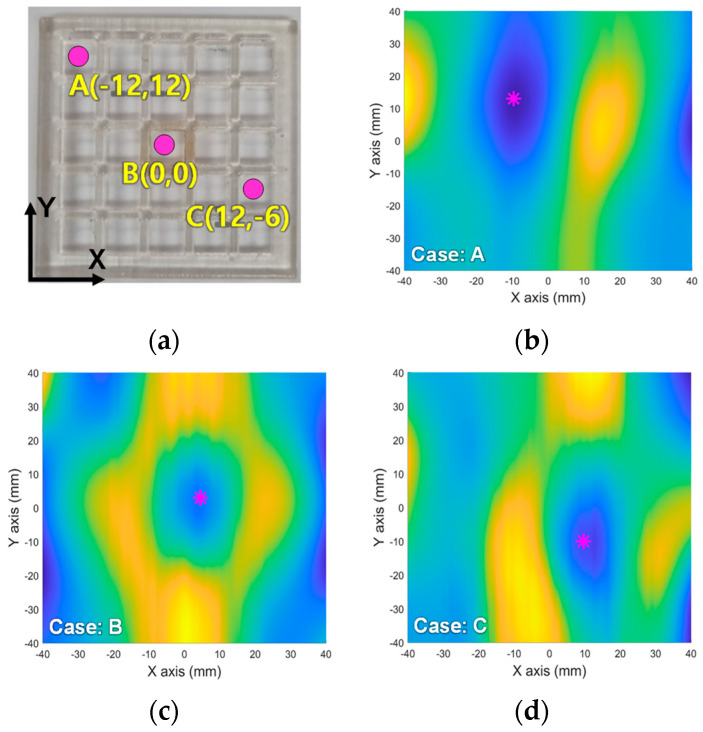
Two-dimensional magnetic particle image of MNPs (0.3 mg) on the XY plane (ROI of 80 × 80 mm^2^). (**a**) A 1 mm grid plate phantom; (**b**) a 2D image of MNPs located at A(−12, 12); (**c**) a 2D image of MNPs located at B(0,0); (**d**) a 2D image of MNPs located at C(12, −5). In (**b**–**d**), the red stars describe the predicted location of MNPs.

Two-dimensional localization experiments were carried out using a MNP container (Hexahedral 3 × 3 × 4 ${mm}^{3}$). The mass of the MNPs inserted into the container to effectively evaluate imaging performances was 0.3 mg. The sampling time and velocity of the robotic arm were the same as those used in one-dimensional performance tests, and it moved along the zigzag path on the XY plane. The MNPs were placed at the three positions of A(−12, 12), B(0, 0), and C(12, −6). Figure 8 shows the localization results for three cases. The average scanning time was 50 s and the data of the MNP response signals formed the basin in the XY plan. Through the GDM, the valley points were estimated as the red stars on Figure 8b,d. Actually, the predicted valley points for each case were (−9.6, 13) for A, (4.5, 3.0) for B and (9.6, −10.0) for C, respectively. As the results, each localization error was (2.4, 1), (4.5, 3.0) and (2.4, 4), and the root mean square error (RMSE) of two-dimensional localization, averaged across three cases, was evaluated as 4.1 mm. Consequently, the proposed system demonstrated its ability to both visualize the location of the MNPs and convert the MNP location into data.

## 4. Conclusions

This paper presented a newly proposed coil structure for localizing MNPs to achieve precise TDD, distinguishing it from conventional MPI systems. The proposed system was constructed as a simplified coil structure combined with the permanent magnet for magnetizing MNPs; hence, it did not rely on FFPs or FFLs, which are commonly used in conventional MPI systems for imaging and localizing MNPs. The localization performance of MNPs in the proposed system was verified through several one- and two-dimensional imaging tests. Experimentally, the minimum detectable mass of MNPs was 0.3 mg, and the RMSE of the localization error was evaluated at 4.1 mm. One-dimensional detection tests in particular highlight not only the system’s sensitivity to MNPs but also its potential for quantitative analyses of different MNP quantities within the detection range. In conclusion, the proposed system offers an alternative solution to the challenges faced by conventional MPI, as mentioned in the introduction. Future research will prioritize the development of the system’s quantification functionality to improve its reliability and obtain more well-defined spatial images of MNPs. Furthermore, to increase applicability of the proposed system, it is necessary to acquire the three-dimensional location of MNPs in real time. The proposed system will be implemented to localize MNPs in three dimensions by scanning multiple planes and integrating the location data from each plane. Finally, it will demonstrate the ability to perform both the actuation and imaging of MNPs in a three-dimensional space for practical TDD applications.

## Figures and Tables

**Table 1:** Design specification of an open-type RF device.

Coil Type	Inner Diameter (mm)	Outer Diameter (mm)	Height (mm)	Turns	Wire Diameter (mm)
${\mathrm{Rx}}_{\mathrm{Cali}}$	40	80	20	450	0.5
${\mathrm{Rx}}_{\mathrm{Col}}$	40	80	5	150	0.5
Tx	43	113	20	783	0.8

## Data Availability

Data is contained within the article.
